# *Badister* Clairville 1806: A new species and new continental record for the nominate subgenus in Amazonian Perú (Coleoptera, Carabidae, Licinini)

**DOI:** 10.3897/zookeys.147.2117

**Published:** 2011-11-16

**Authors:** Terry L. Erwin, George E. Ball

**Affiliations:** 1 Hyper-diversity Group, Department of Entomology, MRC-187, National Museum of Natural History, Smithsonian Institution, Washington, P.O. Box 37012, DC 20013-7012, USA; 2 Department of Biological Sciences, University of Alberta, Edmonton, Canada

**Keywords:** Taxonomy, classification, Licinina, *Baudia* Ragusa 1884, species key, Neotropical Region, Varzea, Igapó

## Abstract

*Badister (Badister) amazonus* **sp. n.** is described from Perú, Loreto, 1.0 km SW Boca del Rio Samiria, Vigilante Post 1, 130m, “04°40.5'S, 074°18.9'W" its type locality. It is known also from two other localities in Loreto Department, Perú, in both the Varzea and Igapó river systems. This new species is sufficiently different that a new informal higher taxon, the *amazonus* species complex, is recognized. An updated key to the Western Hemisphere species of subgenus *Badister* is provided.

## Abstract (es)

*Badister (Badister) amazonus* especie nueva, se describe de Perú, Loreto, 1.0 km SW Boca del Rio Samiria, Vigilante Post 1, 130m, “04°40.5'S, 074°18.9'W", su localidad tipo. Se conoce también de dos otras localidades en el Departamento de Loreto, Perú, en los sistemas de los ríos Varzea and Igapó . Esta nueva especie es suficientemente diferente que un nuevo taxón más elevado, el complejo de especies *amazonus*, se reconoce. Se presenta una clave actualizada de las species del subgénero *Badister* para el Hemisferio Occidental.

## Introduction

Unlike the movements of the comic book character Superman, taxon range extensions rarely come to our notice in “leaps and bounds.” And, almost never does a newly discovered member of a subgenus show up on a new continent quite apart from its well recorded and quite distant range. Ball (1959) noted that the southernmost occurrence of the subgenus *Badister* (*s. str.*) Clairville 1806 in the Western Hemisphere was in Oaxaca, México “on or near the edge of the Neotropical Region.” Since then, Ball (1992) also recorded *Badister (Baudia) reflexus* LeConte from Quintana Roo, *Badister (s. str.) flavipes mexicanus* Van Dyke from Chiapas and Tabasco, México, somewhat south and east of Oaxaca, and a single specimen of subgenus *Baudia* Ragusa 1884 from Tucumán, Argentina. In the collection at the Strickland Museum, University of Alberta, Edmonton, Canada, an undescribed brachypterous species from Veracruz, México is represented and at the National Museum of Natural History, Washington, DC, seven undescribed species of the subgenus *Baudia* are represented from various places in southern México, Belize, Honduras, and Costa Rica. Before the discovery reported in this paper, the southern limits in the Western Hemisphere of the subgenus *Badister* was known to be in Chiapas and that of subgenus *Baudia* in the Province of Puntarenas, Costa Rica, and according to Ball (1992) in Argentina (noted above). Another licinine monobasic genus, (the rather distantly related though con-subtribal) *Eutogeneius* Solier 1849, is known from Chile. Between that country and Argentina and Costa Rica, nothing had been recorded for Licinini and the tribe was considered amphi-tropical.

To the senior author’s great surprise on the night of May 21, 1990, at Cocha Shinguito, Loreto Department, Perú, he encountered two adult members of a relatively large species of *Badister* in a swamp forest at the edge of a standing pond in wet leaf litter overlaying gray clay soil (Kaolin). On a subsequent expedition to the same watershed the following year with the junior author, many more adults were encountered at two localities in low damp or wet places near the mouth and at the midpoint of the Río Samiria in the Pacaya-Samiria National Reserve (noted without discussion by Ball 1992). This river lies in a vast subsidence zone on the western edge of the Amazon Basin. The watershed is composed of “black water;” this includes the Igapó main river, many smaller 2^nd^ order streams, and hundreds of small to large oxbow lakes (“cochas” in Spanish and on some country maps). On the subsequent expedition mentioned above, this species was again found (September 2, 1991) along a major white water river, the Río Ucayali. We take this opportunity, as part of the Bell Fest Symposium volume, to describe the new species, its way of life as far as is known, and its place in a biogeographic context of the Genus *Badister* Clairville.

Ross and Joyce Bell, to whom this volume is dedicated, have lived in Vermont, USA, throughout their long and productive careers in taxonomic studies of Rhysodini and other carabids. Coincidentally, they have lived in an area of maximum species richness for *Badister*, with no less than five species occurring in their surroundings.

## Specimens and methods

Included in this study are a total of 58 specimens from the National Museum of Natural History, Washington, DC (NMNH, Terry L. Erwin, Curator) and 14 specimens from the Strickland Museum, University of Alberta, Edmonton, Canada (UASM, George Ball and Danny Shpeley, Curators). Some of these specimens will be deposited in the California Academy of Sciences (CAS, David H. Kavanaugh, Curator), the Carnegie Museum of Natural History (CMNH, Robert Davidson, Collection Manager), the Museum of Comparative Zoology (MCZ, Philip Perkins Collection Manager), and the holotype in the Museo de Universidad de San Marcos, Lima, Perú (MUSM, Gerardo Lamas, Curator).

Methods and species concepts follow those previously described (Ball 1959; Erwin and Kavanaugh 1981; Kavanaugh and Erwin 1991). The species validation and diagnosis format follows as closely as possible that suggested in Erwin and Johnson (2000). Measurements of length (ABL, SBL) and width (TW) follow those of Ball (1972) and Kavanaugh (1979): ABL (apparent body length), measured from apex of labrum to apex of longer elytron; SBL (standardized body length), equals the sum of the lengths of the head (measured from apex of clypeus to a point on midline at level of the posterior edge of compound eyes), PL (pronotal length ), measured from apical to basal margin along midline, and LE (elytron length), measured from apex of scutellum to apex of the longer elytron; and TW (total width), measured across both elytra at their widest point with suture closed.

Terms used for the pygidial gland system were taken from Will et al. (2000). For the ovipositor sclerites, terms used are those of Liebherr and Will (1998). For explanation of orientation of the surfaces of gonocoxite 2, see Ball and Shpeley (2009: 92). Note also that Ball (1959) referred to gonocoxite 2 as the “stylus” and its blade as the “digitus.” The terms for the parts of the male genitalia are either standard among carabidologists, or are taken from Ball and Shpeley (2009: 92). Attributes of the abdominal ventral sterna are referred to using the numbering system generally accepted in Carabid studies, i.e., the sternum divided medially by the hind coxae is sternum II (the first being hidden) and the last visible is sternum VII (Liu et al. 2011).

**Figure 1.**
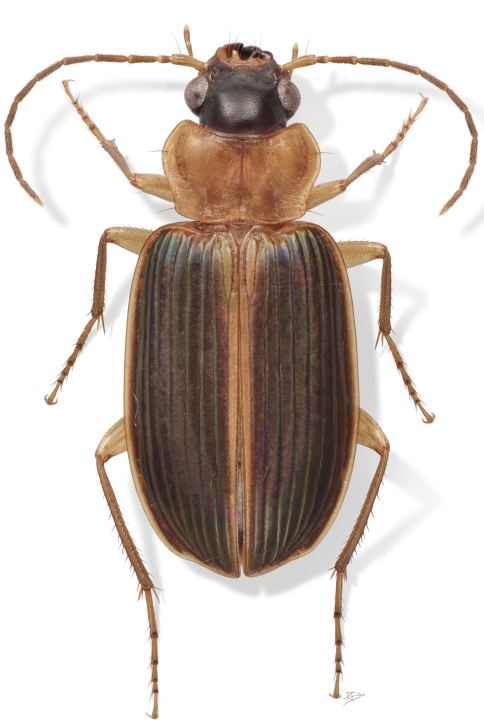
*Badister amazonus* sp. n., female, ADP051544; type locality. Habitus, dorsal aspect. ABL = 7.13mm.

The habitus image (Fig. 1) of the adult beetle portrays most of the character states referred to in the key provided. Male and female genitalic digital photo-illustrations are standard for descriptive taxonomy of carabid beetles. The images of the adult and its parts were made with a Visionary Digital^TM^ high resolution imaging system. Figure captions include an ADP number, which is a unique identification number for the specimen that was illustrated or imaged and links the specimen and associated illustrations and/or image to additional information in electronic databases at the NMNH.

Throughout this paper, a hyphen (-) is used for its normal purpose; a dash (–) is used to indicate a continuum between entries (e.g., between measures, altitudinal ranges, Figure numbers or letters, or across months). Geographical data are presented for the new species based on all known specimens available at the time of manuscript preparation. Georeferences have been determined from locality information provided on specimen labels. Latitude and longitude are reported in decimal degrees. A distribution map is provided for the species (Fig. 7). Here, an English vernacular name is proposed, as vernacular names are becoming increasingly needed in conservation and/or agricultural and forestry applications, as well as for the Encyclopedia of Life (www.eol.org).

## Accounts of taxa

### Fast-walking Beetles Badister

Clairville, 1806

http://species-id.net/wiki/Badister

#### Type species.

*Carabus bipustulatus* Fabricius, 1792: 161

**Number of described Western Hemisphere species:** 16

#### Taxonomy.

Stable, although several recently collected undescribed species need to be treated. Adelphotaxon: not determined.

#### Geographic Distribution.

Members of this genus occur in the Antillean sub-region of the Neotropical Region, Amazon Basin of Perú, the Holarctic Region, Afrotropical Region, in the Cape sub-region, and on the island of Madagascar (Ball 1959), Oriental Region eastward to Java, and the Australian Region, including New Guinea and eastern Australia (Darlington 1968: 15).

#### Habitat.

Wet and moist areas including shaded margins of vernal pools in deciduous forests, open margins of alkaline lakes, secondary floodplains of river banks, at eutrophic marshes, pond and lakes, in disturbed places such as gravel pits, vacant lots, pastures, and cultivated fields (e.g., barley).

#### References.

(Ball (1959, 1992), Jeannel (1942), Larochelle and Larivière (2003).

#### Diagnostic combination.

**(modified from Ball 1959)**.— With character states of subtribe Licinina (Ball 1992: 347, in key), and the following: Length not exceeding 10 mm, body glabrous, except for certain constant setae mentioned below; mandibles thick, either right or left mandible with deep transverse notch in dorsal surface, notch preceded proximally by a prominent boss; tibial spurs finely serrulate; antennomeres 3–11 densely setose.

#### Description.

**Figure 2. F2:**
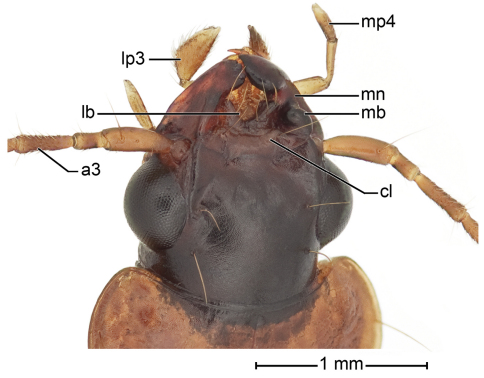
*Badister amazonus* sp. n., dorsal aspect, female, ADP051544; type locality. Head, including eyes, antennomeres 1–3, mouthparts, and anterior part of pronotum. Legend: **a3**, antennomere 3; **cl**, clypeus; **lb**, labrum; **lp3**, labial palpomere 3; **mb**, mandibular boss; **mn**, mandibular notch; **mp4**, maxillary palpomere 4.

**Head** (Fig. 2) with two supraorbital setigerous punctures per eye, frontal impressions usually shallow, basin-like. Clypeus (**cl**) markedly and slightly asymmetrically emarginate, not divided into two portions by the emargination, deflected ventrally, forming an angle of somewhat less than 90 degrees with the front, bearing a single long seta on each side, each of which originates in a broad approximately triangular pit. Eyes prominent. Antenna with complex variety of sense organs beginning on antennomere 3 (**a3**). Labrum (**lb**) about 2/3 as wide as clypeus, deeply bilobed, cleft anteriorly almost extended to base, with three setae on each lobe. Mandibles very short and obtuse, terebra markedly reduced, apparently edentate; either right or left mandible deeply notched (**mn**), preceded by a prominent swelling, or boss (**mb*)***. Maxillary palpi long and slender, palpomere 4 (**mp4**) fusiform, slightly broader than palpomeres 1–3, narrowly truncate apically. Labial palpus with palpomere 2 bisetose; palpomere 3 (**lp3**) securiform, distal margin obliquely truncate.

**Prothorax. Pronotum** (Fig. 1) wider than head, more or less cordate, a pair of setigerous punctures on each side, one of the pair at posterior lateral angle, the other just inside lateral margin about half way between base and apex; narrowly margined laterally, hind angles obtuse, slightly to markedly rounded. Proepisternum with microsculpture mesh pattern longitudinal; microlines fine, transverse on remaining lateral and ventral thoracic sclerites. Prosternum with apex of intercoxal process feebly margined, asetose.

**Pterothorax.** Metepisternum elongate, the outer margin about 1.5 times greater in length than the anterior margin, posterior margin about 0.75 times anterior margin.

**Figure 3. F3:**
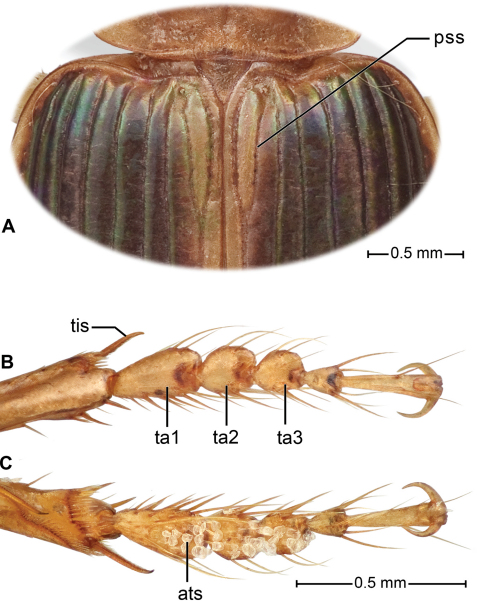
*Badister amazonus* sp. n., dorsal aspect, female, ADP051544; type locality. A. Scutellar region of elytra: (**pss**) parascutellar stria; B–C. Foreleg tarsomeres of male (ADP051713); B. Dorsal aspect, C. ventral aspect; **ats,** articulo-seta; **ta1**, **ta2**, **ta3**, tarsomeres 1–3.

**Elytra.** Oblong, wider than pronotum at widest point, apical margin entire or very slightly sinuous, interneurs fine, parascutellar interneur (Fig. 3A, **pss**) long, joined or not to apical portion of interneur 1; intervals flat to slightly convex with two discal punctures on interval 3, adherent to interneur 2. Lateral marginal (umbilical) series of 15–18 setae, concentrated and narrowly spaced in anterior and posterior thirds, with few widely spaced setae medially.

**Hind wings**. Macropterous or brachypterous. Venation not studied.

**Legs.** Slender; tibial spurs very finely serrulate; posterior tarsi very slender, dorsal face dull and medially feebly sulcate; anterior tarsi of male (Fig. 3B–C) with tarsomeres 1–3 markedly or slightly dilated, with extensive or limited patch of adhesive articulo-setae ventrally (Fig. 3C) (also, see Stork 1980: 195, 290, for detailed description of adhesive setae of *Badister bipustutulatus* (Fabricius))

**Abdominal sterna.** Surfaces with very fine, transverse microlines, very close together, surface quite iridescent. Abdominal sternum VII with two setigerous punctures on posterior margin in male, four in female, apical margin more rounded in the male, more truncate in the female.

**Figure 4. F4:**
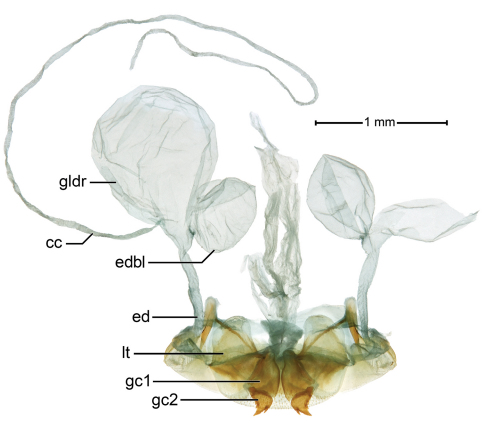
*Badister amazonus*sp. n., dorsal aspect, ADP051543; type locality. Pygidial (defense) gland system, Legend: **cc**, collecting canal; **ed**, efferent duct; **edbl**, efferent duct, basal lobe; **gldr**, pygidial gland reservoir. Ovipositor sclerites, Legend: **gc1,** gonocoxite 1; **gc2,** gonocoxite 2; **lt**, laterotergite

Pygidial glands as in Fig. 4, showing a short efferent duct(**ed**),a large gland reservoir(**gldr**),a long narrow collecting canal (**cc**),and basal lobe of efferent duct (**edbl**).

**Figure 5. F5:**
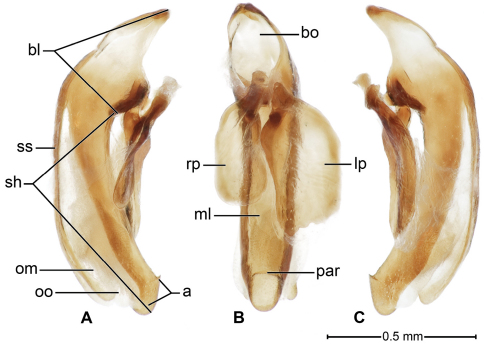
*Badister amazonus* sp. n., ADP127149; type locality. Male genitalia, median lobe, and parameres, A - left lateral, B - dorsal, and C- right lateral aspects. Legend: **a**, apical area; **bl**, basal lobe; **bo**, basal orifice**; lp**, left paramere; **ml**, median lobe; **om**, ostial membrane; **oo**, ostial opening; **par,** preapical ridge**; rp**, right paramere; **sh**, shaft; **ss,** dorsal sclerotized strip.

**Male genitalia** (Fig. 5A-C)**.** Median lobe moderately arched in typical subgenus, straighter in subgenus *Baudia*, (see Figs 134–155 in Ball 1959); basal bulb somewhat reflexed, sides not emarginate, dorsally mostly membranous, with two or three long sclerotized strips.

**Figure 6. F6:**
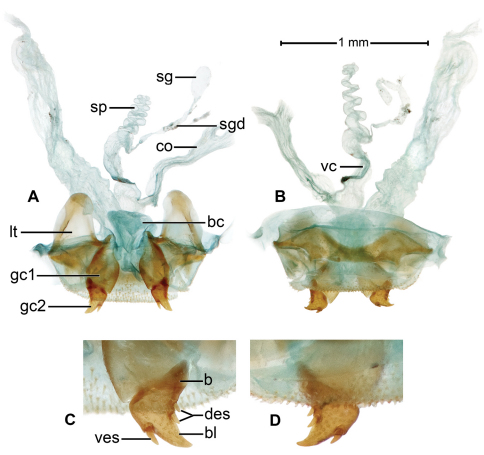
*Badister amazonus* sp. n. ADP127151; type locality. Female genital tract. A. Ventral aspect, B. Dorsal aspect: Legend, **b,** base of gonocoxite 2; **bl**, blade of gonocoxite 2; **des**, dorsal ensiform seta; **gc1**, gonocoxite 1; **gc2**, gonocoxite 2; **lt**, laterotergite; **ves,** ventral ensiform setae**.** Ovipositor. C. Ventral aspect, D. Dorsal aspect: Legend, **bc,** bursa copulatrix; **co,** common oviduct; **sg,** spermathecal gland; **sgd**, spermathecal gland duct; sp, spermatheca. dorsal aspect; **vc**, villous canal.

**Ovipositor and female reproductive organs** (Fig. 6A–D). Gonocoxite 2 (**gc 2**)falcate, base (**b**) about as long as blade (**bl**), latter relatively short, pointed distally; margins with several ensiform setae; with or without short preapical nematiform setae. Reproductive organs standard for Carabidae, with bursa copulatrix, common oviduct, spermatheca, and spermathecal glands (for details, see this topic below, in description of *Badister amazonus*, n. sp.).

### Subgenus Badister

Clairville, 1806

Badister Clairville, 1806: 90
Amblychus Gyllenhal, 1810: 74

#### Diagnosis.

In addition to the features presented in the generic diagnosis and description above, all of the Western Hemisphere species of the typical subgenus have the right mandible deeply notched (Fig. 2, **mn**), and a row of setae on each latero-ventral margin of tarsomere 5. Another useful attribute in distinguishing the subgenera is the relative length of the hind tarsi. In adults of *Badister* and *Trimorphus*, the posterior tarsi are more than 3/4 the length of the hind tibiae; in *Baudia*, the hind tarsi are perceptibly relatively shorter.

#### Geographic Distribution.

(**Western Hemisphere**).—The known range of this subgenus includes Amazonian Perú in South America, Middle American countries of Costa Rica, Honduras, Belize and México, and the North American countries of USA and Canada.

##### Key to the Western Hemisphere Species of Subgenus *Badister* Clairville, 1806

(Modified from Ball 1959)

**Table:** 

1	Anterior margin of proepisternum and lateral margins of prosternum distinctly rugose; maxillary palpomere 3 distinctly shorter than palpomere 4; head and pronotum black, shining surface of pronotum smooth without microlines; head with mesh pattern isodiametric, microlines shallow, very fine, hardly discernible at magnification of 18 dia; elytra rufo-piceous, striae deep, finely punctate, intervals narrow, convex	*Badister notatus* Haldeman, 1843
1’	Anterior margin of proepisternum and prosternum not rugose, but rugulose in some specimens; maxillary palpomere 3 distinctly shorter than or subequal to palpomere 4; surface of head and pronotum with mesh pattern isodiametric, surface dull, or mesh pattern transverse, markedly iridescent; elytral striae shallow, intervals relatively broad and flat	2
2(1’)	Elytra not iridescent, microlines relatively far apart, in form of obvious sculpticells, as seen under magnification of 54 dia	*Badister obtusus* LeConte, 1878
2’	Elytra markedly iridescent, microlines relatively close together, not in form of obvious sculpticells, as seen under magnification of 54 dia	3
3(2’)	Elytra either concolorous or somewhat paler at base and/or paler marginally and interval 1, but not markedly bicolored	4
3’	Elytra markedly bicolored	9
4(3)	Elytra black	5
4’	Elytra piceous to rufescent	8
5(4)	Pronotum flavotestaceous	*Badister amazonus*, sp. n.
5’	Pronotum black	6
6(5’)	Legs rufous or darker	*Badister ferrugineus anthracinus* LeConte, 1859
6’	Legs testaceous	7
7(6’)	Lateral margins of pronotum rounded evenly into lateral portion of basalmargin, the posterior angles thus broadly rounded	*Badister flavipes flavipes* LeConte, 1853
7’	Lateral margins of pronotum sinuate posteriorly, in form of a shallow notch in front of posterior marginal setiferous punctures, posterior angles obtuse, narrowly rounded	*Badister flavipes mexicanus* Van Dyke, 1945
8(4’)	Pronotum with base relatively narrow (W base/W apex = 0.93–1.07, W base/L pn 0.96 –1.10), antennae testaceous to rufo-testaceous throughout	*Badister flavipes laticeps* Blatchley, 1910
8’	Pronotum with base relatively broad (W base/W apex = 1.05–1.16, W base/L pn= 1.18 –1.33).Antennomeres 4–7 rufo-piceous or darker, distinctly darker than rest of antenna	*Badister ferrugineus ferrugineus* Dejean, 1831
9(3’)	Pronotum orange-testaceous	10
9’	Pronotum black	11
10(9)	Metepisternum orange-testaceous, or at least paler than metasternum, maxillary palpomere 3 distinctly shorter than palpomere 4; scape of antenna uniformly testaceous throughout	*Badister pulchellus* LeConte, 1848
10’	Metepisternum black, or at least as dark as metasternum, maxillary palpomere 3 only very slightly shorter than or equal in length to the palpomere 4	*Badister neopulchellus* Lindroth, 1954
11(9’)	Sides of pronotum distinctly sinuate before hind angles, the angles therefore prominent	*Badister vandykei* Ball, 1959
11’	Sides of pronotum not sinuate before hind angles, angles obtusely rounded	12
12(11’)	Pronotum relatively long and slender (W pn wp/L pn= 1.15–1.35), elytral interval 1 in basal 1/3 not darker than intervals 2–8	*Badister elegans* LeConte, 1880
12’	Pronotum broadly cordate, relatively short and broad (W pn wp/L pn = 1.40–1.55), elytral interval 1 black, or at least distinctly darker than intervals 2–8, at least in basal 1/3	*Badister maculatus* LeConte, 1853

## Species Account

### Badister amazonus

Erwin & Ball sp. n.

urn:lsid:zoobank.org:act:D953E83C-2809-4DE7-91F2-40451DEBFDEA

http://species-id.net/wiki/Badister_amazonus

Figures 1 [Fig F2] [Fig F3] [Fig F4] [Fig F5] [Fig F6] –7

#### Holotype.

**Perú**, Loreto, 1.0 km SW boca del Rio Samiria, Vigilante Post 1, 130m, “04°40.5'S, 074°18.9'W" 31 August 1991 (T.L. Erwin & M.G. Pogue)(NMNH: ADP051824, male).

#### Derivation of specific epithet.

 The epithet “*amazonus*” is a singular Latinized masculine noun in apposition, based on the name of the area in which these beetles were found.

#### Proposed English vernacular name.

 Amazon Fast-walking Beetle.

#### Diagnosis.

 With the attributes of the subgenus *Badister*, as described by Ball (1959) and as noted above, and large-sized for the genus (mean SBL more than 6.1 mm for both males and females of *Badister amazonus*; mean SBL less than 5.9 mm for males and females of all other Western Hemisphere species samples). Adults with black head and elytra, the elytron with flavotestaceous base, sutural interneur, lateral margin, and epipleuron, and shiny throughout; prothorax and venter flavotestaceous; appendages testaceous. Microsculpture mesh pattern of head and pronotum isodiametric, surface luster somewhat dull; of elytron, very finely etched transverse lines, surface shiny iridescent. Labial palpomere 3 (Fig. 2, **lp3**) swollen and triangulate (distal margin obliquely truncate) with a very small pointed knob at apex. Pronotum with lateral margin moderately explanate in anterior half, broadly so posteriorly. Male tarsomeres 1–3 (Fig. 3B, 3C) slightly widened, with rows of adhesive vestiture on ventral surface narrow, confined to anterior half).

#### Description.

**Figure 7.**
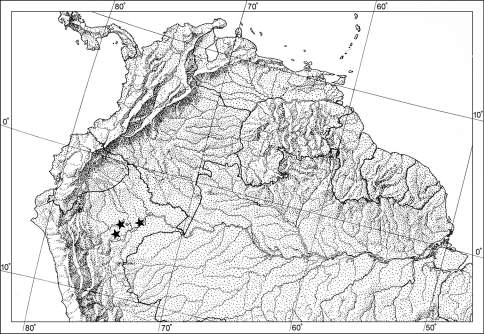
Distribution dot map for known localities of *Badister amazonus* sp. n.. along the rivers Samiria and Ucayali (★).

 (Fig. 1, 2, 3A–C, 4, 5A–C, 6A–D, 7). *Size*: Moderately large for the subgenus; ABL = 6.2–7.9mm, SBL = 5.36–6.73mm, EW (maximum width) 2.55–3.43mm, LP = 0.97–1.22mm, WP = 1.71–1.91 mm, LE = 3.86–4.84mm. *Color*: See Diagnosis above. *Luster*: See Diagnosis above. **Head** (Fig. 2): clypeus (**cl**) medially depressed, unisetose laterally, no clear demarcation from frons. Frons markedly depressed, apically with depression extended to eye carina, posteriorly somewhat concave with slight swellings medially raised to occiput; occiput shallowly domed; neck broad. Eye moderately convex; gena short and flat. Antennae and mouthparts typical for genus (see above for details) .

**Prothorax. Pronotum** (Fig. 1, 2) markedly broad, about twice as long as head (mean LP/LH: 1.683 for males , 1.846 for females), cordiform, margin narrowly explanate with seta at anterior third; base deeply and bilaterally depressed; hind angle slightly obtusely produced and setose; moderately broader than long (mean W/L: 1.71 for males, 1.91 for females); surface medially smooth, slightly rugose laterally medial to explanation.

**Pterothorax.** Normal for genus.

**Elytra**. Elytron about same width as head across eyes (mean WH/WE: 0.998 for both sexes), moderately convex, intervals moderately convex and slightly more so laterally, interval 3 bisetose with each setigerous puncture adherent to interneur 2.

**Hind wings**. Macropterous.

**Legs.** (Fig. 1). Overall, normal for subgenus. Male front tarsus (Fig. 3B, 3C) with tarsomeres 1–3 slightly dilated and each ventrally (Fig. 3C) with 2–4 rows of white articulo-setae.

**Abdominal sterna** with normal setation for genus.

**Male genitalia** (Fig. 5A–5C). Median lobe (**ml**) with basal lobe (**bl**) about half length of shaft (**sh**), basal opening (**bo**) large. Shaft slender, curved ventrally, dorsally membranous (**om**) except for two long sclerotized strips (**ss**) extended from basal lobe to ostial opening (**oo**); in ventral aspect tapered toward rather broadly rounded apex, preapically with prominent preapical ridge (**par**), in lateral aspect, ridge a prominently projected point. Parameres (**lp, rp**)broad, apices subtruncate left paramere **(lp**) longer than right paramere (**rp**) about three quarters length of shaft (measured in left lateral aspect). Internal sac with apical ring of rather densely distributed microtrichia, without preapical spines.

**Female genitalia.** (Fig. 6A–D). Ovipositor with broad laterotergite (**lt**) and two gonocoxites (**gc 1, gc 2**); gonocoxite 1 asetose; gonocoxite 2 falcate, base (**b**) large, broad, blade (**bl**) rather short, with two dorsal ensiform setae (**des**), and one ventral ensiform seta (**ves**), all ensiform setae short; without ventral preapical nematiform setae. Reproductive tract (Fig. 6A, 6B) proximally with short, broad bursa copulatrix (**bc**), continuous at its distal end with common oviduct (**co**) and long spermatheca (**sp**), latter coiled distally; villous canal (**vc**) extended from near base of spermatheca well up common oviduct; spermathecal gland (**sg**) bulbous; spermathecal gland duct (**sgd**) long, slender, attached to spermatheca at base of its coiled portion.

Markedly similar to homologous structures in *Badister (Baudia) reflexus* LeConte (Will 1998: 99, Fig. 8), which differ as follows: spermatheca with basal uncoiled portion more extensive; distal coiled portion short; spermathecal gland elongate digitiform.

**Classification.** Ball (1959: 194–195) arranged the species of subgenus *Badister* in two “complexes” characterized principally by features of the male genitalia and ovipositor gonocoxite 2. The combination of these features in *Badister amazonus* fits neither complex. Accordingly, we propose here the monobasic *amazonus* complex, characterized as follows: median lobe of male genitalia dorsally with two long sclerotized strips, internal sac without sclerotized plates or spines; gonocoxite 2 of ovipositor relatively broad basally, ensiform setae relatively short; nematiform setae absent. Additional diagnostic features are: male fore tarsomeres 1–3 (Fig. 3B) relatively slender, not much wider than tarsomeres 1–3 of middle and hind tarsi, fore tarsomere 1 longer than wide, ventral adhesive setae (Fig. 3C) concentrated in anterior half of each tarsomere.

**Dispersal potential**. These beetles are fully macropterous and are probably capable of flight; they are fast walkers. They also are climbers on grasses (*Paspalum* spp.).

**Way of life**. Adults are associated with water: in open grassy marshes, on beaches of black and white water rivers, and in forest swamps at the edges of standing black water ponds in leaf litter on grey clay soil (Kaolin). They are active at night and take cover in the day among grass stems and under flood debris. They are active in May and August–September; no teneral adults were encountered in these months.

**Other specimens examined**. **Perú,** Loreto, SW bank of Rio Ucayali, 90m, 4.2089°S, 73.3657°W, 2 September 1991, (T.L. Erwin)(NMNH: ADP051862, female); Pacaya-Samiria National Reserve, Rio Samiria, Cocha Shinguito, 90m, 5.1775°S, 74.6556°W, 21 May 1990 (T.L. Erwin)(NMNH: ADP093676, 128618, females); 1.0 km SW boca del Rio Samiria, Vigilante Post 1, 130m, "04°40.5'S, 074°18.9'W" 31 August 1991–1 November 1991 (G.E. Ball & D. Shpeley)(NMNH: ADP127151, female, 127149, male); 1.0 km SW boca del Rio Samiria, Vigilante Post 1, 130m, "04°40.5'S, 074°18.9'W" 31 August 1991 (T.L. Erwin & M.G. Pogue)(NMNH: ADP051697, 051603, 051336, 051721, 051672, 051743, 051693, 051698, 051722, 051694, 051724, 051692, 051670, 051717, 051718, 051723, 050590, 050589, 050605, 051696, 051720 females and ADP051824, 051630, 051719, 051713, 051826, 051691, 051695 males); 1.0 km SW boca del Rio Samiria, Vigilante Post 1, 130m, "04°40.5'S, 074°18.9'W" 31 August 1991 (T.L. Erwin)(NMNH: ADP051592, 051563, 051544, 051583, 051589, 051559, 051590, 051564, 051567, 051569, 051561, 051581, 051585, 051570, 051584, 051565, 051543 females and ADP051562, 051541, 051591, 051542, 051566, 051588, 051560, 051568 males).

Two males and 12 females at UASM with the following label data: /PERU Dpto. Loreto/ 1 km SW Boca del Rio/Samiria 04°40'29"S, 74°18'55"W 130m/marsh, treading/31.VIII.& 1.IX.91 30–91//T. L. ERWIN EXP./Res. Pacaya-Samiria/G.E. Ball & D. Shpeley/collectors 1991//.

**Geographic distribution**. (Fig. 7). This species is currently known from only three localities: two along the Río Samiria, and one along the Río Ucayali, Loreto Department, Perú.

**Notes**. We are not presenting an analysis of variation in body proportions because this species can be separated from all other members of the genus without resorting to such attributes. In regard to the location (latitude/longitude) above for “1.0 km SW del boca Rio Samiria,” Google Earth allows us now to refine what is given on the labels. The actual site is 4.686779°S, 74.336711°W at an elevation of 101m. Also, the female specimen from the Río Ucayali was mistakenly labeled “Río Amazonas.” The river changes names in the vicinity of Iquitos, Perú.

## Concluding statement

Although we have demonstrated that the genus *Badister* in the Western Hemisphere is not amphi-tropical, we are left with many questions. Is it not anomalous that a distinct species, such as *Badister amazonus*, should be found so distant geographically from its taxonomically close relatives in southern México, and yet be so structurally similar to them? One might expect that this seeming isolate would be markedly differentiated within the subgenus in which it has been placed, in other words, some sort of a relict; however, that seems unlikely. Rather, *Badister amazonus* gives the impression of a taxon that is a comparatively recent arrival in South America, and one could expect it to have a geographical range that extends along northern South American waterways, as well as those of the Amazon Basin. On the other hand, *Badister amazonus* could be a species with a restricted distribution that has undiscovered close relatives along these waterways that, collectively, would close the seemingly extensive gap in the overall range of subgenus *Badister*. Additionally, the occurrence of subgenus *Baudia* in mid-northern Argentina begs the questions: 1) Given that many species of this subgenus are known to be tropical (from southern México to Costa Rica), why has it not been found in the northern South American tropics as it has in subtropical/temperate Argentina? 2) Is the Argentine specimen mislabeled? 3) Is it an invasive species?

So, questions are posed that will be answered by hardy field workers prepared to endure the hardships and risk to life and limb that one faces from unwanted encounters with the crocodilian and reptilian denizens inhabiting the South American wetlands, the home grounds of *Badister amazonus* n. sp. Such exploration is the torch that senior carabidologists pass on to following generations of carabid beetle enthusiasts.

## Supplementary Material

XML Treatment for Badister

XML Treatment for Badister

XML Treatment for Badister amazonus

## Appendix 1

**Table 1.** Tables with measurements and ratios for *Badister amazonus* sp. n. corresponding to same in Ball (1959) for all other western hemisphere species of Badister. All measurements in millimeters.

TOTAL LENGTH (SBL)
	Males	Females
Species amazonus	N	Range	Mean	N	Range	Mean
15	5.667-6.517	6.144	15	5.357-6.73	6.236

MAXIMUM WIDTH
	Males	Females
Species amazonus	N	Range	Mean	N	Range	Mean
15	2.64-3.116	2.942	15	2.554-3.428	3.042

W of HEAD/W of L. ELYTRON
	Males	Females
Species amazonus	N	Range	Mean	N	Range	Mean
15	0.959-1.034	0.998	15	0.927-1.054	0.998

PRONOTUM: WIDTH (at widest part)/LENGTH
	Males	Females
Species amazonus	N	Range	Mean	N	Range	Mean
15	1.713-1.908	1.814	15	1.710-1.911	1.826

LENGTH OF PRONOTUM / LENGTH OF HEAD
	Males	Females
Species amazonus	N	Range	Mean	N	Range	Mean
15	1.477-1.951	1.683	15	1.712-1.957	1.846

## References

[B1] Ball GE (1959) A taxonomic study of the North American Licinini with notes on the Old World species of the Genus *Diplocheila* Brullé (Coleoptera). Memoirs of the American Entomological Society, 16, iv + 1–258.

[B2] Ball GE (1972) Classification of the species of *Harpalus* subgenus *Glanodes* Casey (Carabidae: Coleoptera). The Coleopterists Bulletin 26: 179-204.

[B3] Ball GE (1992) The tribe Licinini (Coleoptera: Carabidae): a review of the genus groups and of the species of selected genera. Journal of the New York Entomological Society 100: 325-380.

[B4] Ball GE Shpeley D (2009) A taxonomic review of the genus *Apenes* (Coleoptera; Carabidae: Lebiini) in the West Indies, with descriptions of new species and notes about classification and biogeography. Annals of Carnegie Museum 78: 81-191. 10.2992/007.078.0201 PMC5672587 29118595

[B5] Blatchley WS (1910) The Coleoptera or beetles of Indiana. Indiana Department of Geology and Natural Resources, Bulletin No. 1, 1386 pp.

[B6] de Clairville J (1806) Schellenberg JR (1806) Helvetische Entomologie oder Verzeichniss der schweizerischen Insekten nach einer neuen Methode geordnet 2: 1–247. Zurich. [French translation]

[B7] Darlington PJ Jr (1968) The carabid beetles of New Guinea Part III. Harpalinae (continued): Perigonini to Pseudomorphini. Bulletin of the Museum of Comparative Zoology 137: 1-253.

[B8] Dejean PFMA (1831) Species général des coléoptères, de la collection de M. le Comte Dejean, 5, viii + 883 pp. Méquignon-Marvis, Paris.

[B9] Erwin TL Johnson PJ (2000) Naming species, a new paradigm for crisis management in taxonomy: Rapid journal validation of scientific names enhanced with more complete descriptions on the internet. The Coleopterists Bulletin 54 (3): 269-278. 10.1649/0010-065X(2000)054[0269:NSANPF]2.0.CO;2

[B10] Erwin TL Kavanaugh DH (1981) Systematics and zoogeography of *Bembidion* Latreille: I. The *carlhi* and *erasum* groups of western North America (Coleoptera: Carabidae, Bembidiini). Entomologica Scandinavica Supplement 15, 33–72.

[B11] Fabricius JC (1792) Entomologica systematica. Hafniae, 1: 330 + 538 pp.

[B12] Gyllenhal L (1810) Insecta Suecica descripta. Classis 1. Coleoptera sive Eleuterata. Tomi 1, Pars II, xx + 660 pp. F.J. Leverentz, Scaris.

[B13] Haldeman SS (1843) Descriptions of North American species of Coleoptera, presumed to be undescribed. Proceedings of the Academy of Natural Sciences of Philadelphia 1: 298-304.

[B14] Jeannel R (1942) Coléoptères carabiques. Deuxième partie. Faune de France 40. Librairie de la Faculté des Sciences, Paris, 573–1173.

[B15] Kavanaugh DH (1979) Studies on the Nebriini (Coleoptera: Carabidae), III. New Nearctic *Nebria* species and subspecies, nomenclatural notes, and lectotype designations. Proceedings of the California Academy of Sciences 42: 87-133.

[B16] Kavanaugh DH Erwin TL (1991) The tribe Cicindini Bänninger (Coleoptera: Carabidae): Comparative morphology, natural history, and reclassification. Proceeding of the Entomological Society of Washington 93 (2): 356-389.

[B17] Larochelle A Larivière M-C (2003) A natural history of the ground-beetles (Coleoptera: Carabidae) of America north of Mexico. Pensoft, Sofia-Moscow, 583 pp.

[B18] LeConte JL (1853) Notes on the classification of the Carabidae of the United States. Transactions of the American Philosophical Society (Series 2) 10: 363–403.

[B19] LeConte JL (1878) Descriptions of new species. In: Hubbard HG Schwarz EA (Eds). The Coleoptera of Michigan. Proceedings of the America Philosophical Society 17: 593–626.

[B20] LeConte JL (1880) Short studies of North American Coleoptera. Tranactions of the American Entomological Society 8: 163-218.

[B21] Liebherr JK Will KW (1998) Inferring phylogenetic relationships within the Carabidae (Insecta, Coleoptera) from characters of the female reproductive tract. In: Ball GE Casale A Vigna-Taglianti (Eds). Phylogeny and classification of Caraboidea (Coleoptera, Adephaga). Proceedings of a Symposium (28 August 1996, Florence, Italy) XX International Congress of Entomology. Atti, Museo Regionali di Scienze naturali, Torino: 107-170.

[B22] Lindroth CH (1954) Random notes on North American Carabidae (Coleopt.). Bulletin of the Museum of Comparative Zoology 3 (3): 117-161.

[B23] Liu Y Kavanaugh DH Shi HL Liang HB (2011) A Key to species of subgenus *Lithochlaenius* (Coleoptera, Carabidae, Chlaeniini, *Chlaenius*), with descriptions of three new species. ZooKeys 128: 15-52. 10.3897/zookeys.128.1804 PMC3175138 21998550

[B24] Ragusa E (1884) Catalogo Ragionato dei Coleotteri di Sicilia. Naturalista Siciliano 7: 1-6.

[B25] Solier AJJ (1849) Coleópteros Pentámeros. In: Gay C (Ed) Historia físico y política de Chile 4 (Zoología), 105–507.

[B26] Stork NE (1980) A scanning electron microscope study of tarsal adhesive setae in the Coleoptera. Zoological Journal of the Linnean Society 68: 175-306. 10.1111/j.1096-3642.1980.tb01121.x

[B27] Van Dyke EC (1945) New species of North American Coleoptera. Pan-Pacific Entomologist 21: 101-109.

[B28] Will KW (1998) A new species of *Diplocheila* Brullé from North America, with notes on female reproductive tract characters in selected Licinini and implications for evolution of the subgenus *Isorembus* Jeannel (Coleoptera: Carabidae: Licinini). Proceedings of the Entomological Society of Washington 100: 95-103.

[B29] Will KW Attygalle AB Herath K (2000) New defensive chemical data for ground beetles (Coleoptera: Carabidae): interpretations in a phylogenetic framework. Biological Journal of the Linnean Society 71: 459-481.

